# Supplementary material for: Identification of Chlorophyll Metabolism- and Photosynthesis-Related Genes Regulating Green Flower Color in Chrysanthemum by Integrative Transcriptome and Weighted Correlation Network Analyses
Source: Genes (Basel). 2021 Mar 21;12(3):449. doi: 10.3390/genes12030449 (PMC8004015; doi:10.3390/genes12030449)
Supplement: Supplementary file 1 [file genes-12-00449-s001.pdf]

Supplementary Materials

Table S1 List of the primers used for the qRT-PCR experiment

gene name	Forward primer	Reverse primer
CmUBI	AGCTGAGCAGACTCCCGATG	AGGCGAATCATCAGTACCAAGT
HEMA1	ACTAGAAACCGTTCCTACAATCC	AAGTCCTGTCATCACTTCCGTC
HEMA2	AGGTCACCGAATGGATGTCTAAGG	CCGCCAATCTTCCTATCAAATCC
GSA1	ATGTATGGGTGTACTGAGGCTAG	AATGTCATTATAGGGTGATGTTAGG
HEMB1	ATTCGGCTGCTCAAAGACAAGTATC	TCACTAGGGCTCACGACATCTGC
HEMC1	ACTCAACCTCTTGCAGATATTGGTG	TTGTCAGGCAGATAAGTTGGGAC
HEMD1	ATCCACTGTTTTGTATCCTGCCTC	GGTAGAAGATTGACCCAAGCACG
HEMF1	CCAGGAGCACCAAGGCAGTG	CTCGCCTCTCATTTCGGTGC
HEMG1	TGAGAAAGATGGGTATTTGTGGGAG	GGTCAAAGAAAGGTATATCAGAAGG
CHLD1	ATTGTTGAAAGGGAAGGAATCAG	CCTCATTACTCTGTTCTTGAAACTGTG
CHLH1	GACTAACCTTGTCGGGAGTCTAAGG	CACCCTCATAGCCACTAGACAGC
CHLI1	TTACAGTGGTTGCTGCCGACC	ATGCTTTAACACCCTCTGTGAGTGC
CHLM1	CTTGAACGGGAAATACGATACGG	GATCATAATAGAATGTCTTGGGTGC
CRD1	TTGAGAACTGGTGCCAAGACG	GAAAGCGGTTCTTTGAGTATCATTC
DVR1	AATTTGAGGGTGAGTTGGTTG	CATCGCCAAACATCACATAAG
PORA1	AATGCAAGAGTTCCATAGACGATAC	AAGCTTGAATCACTTACTACCTGTG
CHLG1	TGGAACCCTTACACCTGACATAG	TGCCAATGCGTAATATGGTTTAC
CAO1	ATCCCTTCACTACTACCACCTCC	ATCCCAGTATCCTTGAAGACCAG
NYC1	TCAGGGGATCGAGTGGTTATTG	CACAAACATCACATGACGTGCC
NYC2	TGATTTGATTTGGTATAGTTGGGTC	CCTGAAAGTAGGAACTCACGAGC
NYC3	TTTGATTTGGTATAGTTGGGTCG	TAACCACACGATCTCCTGAAAGTAG
CLH1	CTAAAGGGATTAGAGGGAAATCTAC	TCATCTTTTATTGCCATTAAATGAC
PPH1	ACTTCGCAGCATGTAACCCTAAC	CATCTTCTCCCAAACAATTTCTATG
PAO1	GGGCATCTGCAATGTTCTTACC	CCATACAAAGAGCAAACCTTGAGAC
PAO2	ATGGCACGAACATATGACTTCAAAC	TAAGCCAATTTCTAAACGCCAGTAC
RCCR1	TTGTAGTCTTCCACCTGATGTCC	ACTTGTGATGTTCAAGGCTCCTC
SGR1	GTCGCTTCACGTTCATTGTCA	GGACCCCAACACTCGACTTT
CmERF	CGTATCTGGCTAGGTTCTTACTCCTC	AGGGCGTCAACTTTAGCTCCAA
CmbHLH	TCGCTGGTTCTGCTTATTTTCA	TTAAATTGCTTCCTTCCTACGC
CmCLO16a	CGTGCTCGGTGGTATTGT	CTGGCTTCATCACTTCCTATCT
CmCOL16b	TCGGTTATGATTCCACGATGA	AGGATTGCTATTCCCAGTCCA

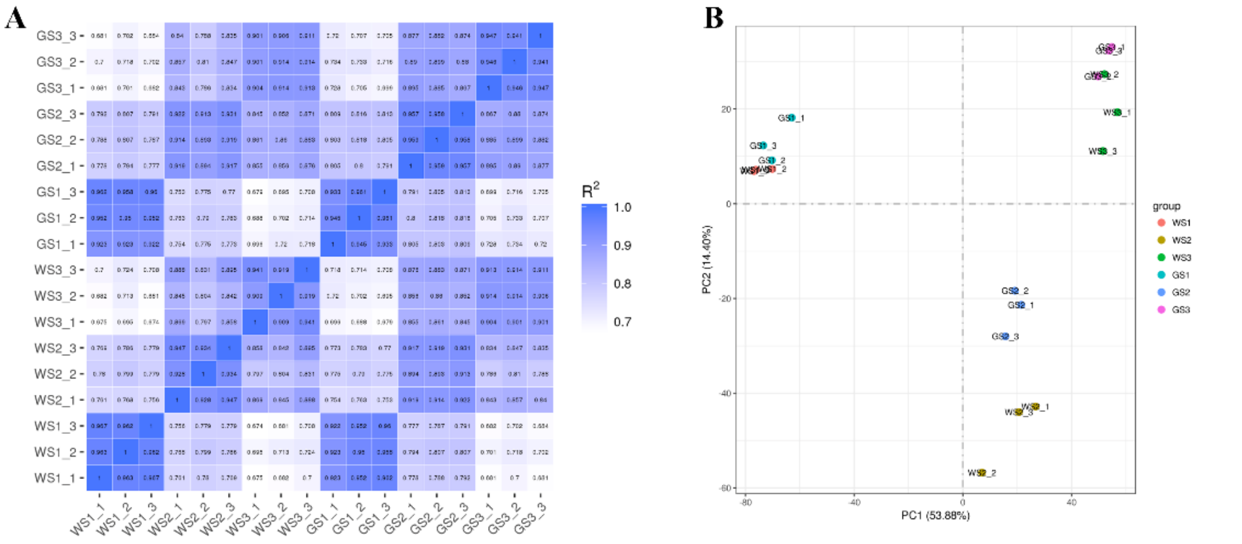

Figure S1 Pearson's correlation and Principal component analysis of gene expressions in each sample of green pools and white pools. **(A)** Pearson's correlation between each sample. **(B)** Principal component analysis of gene expressions in ray florets from green pools and white pools at three stages.

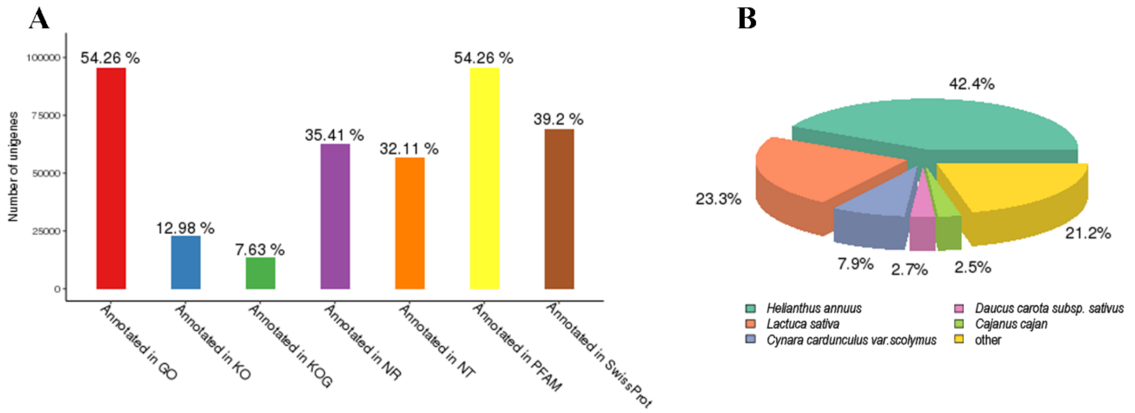

Figure S2 Functional annotations of the transcriptome data. **(A)** Functional annotations of the transcriptome data in seven databases. **(B)** Species distribution of the BLASTX result against the Nr database.

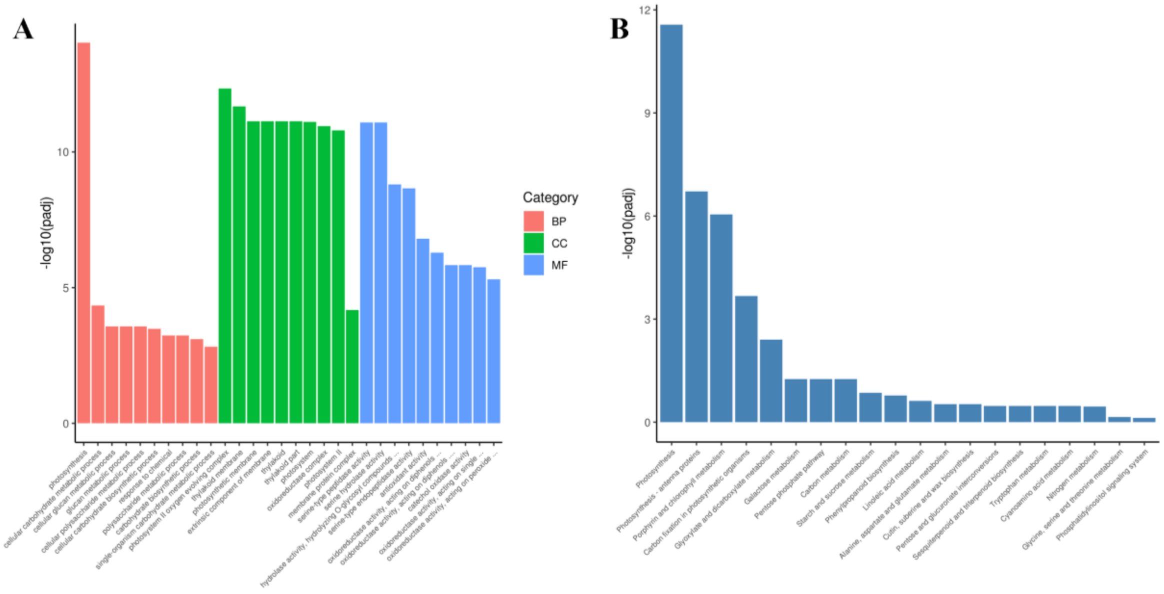

Figure S3 Functional classification of DEGs among different samples. **(A)** GO functional classification of DEGs. **(B)** KEGG functional classification of DEGs.

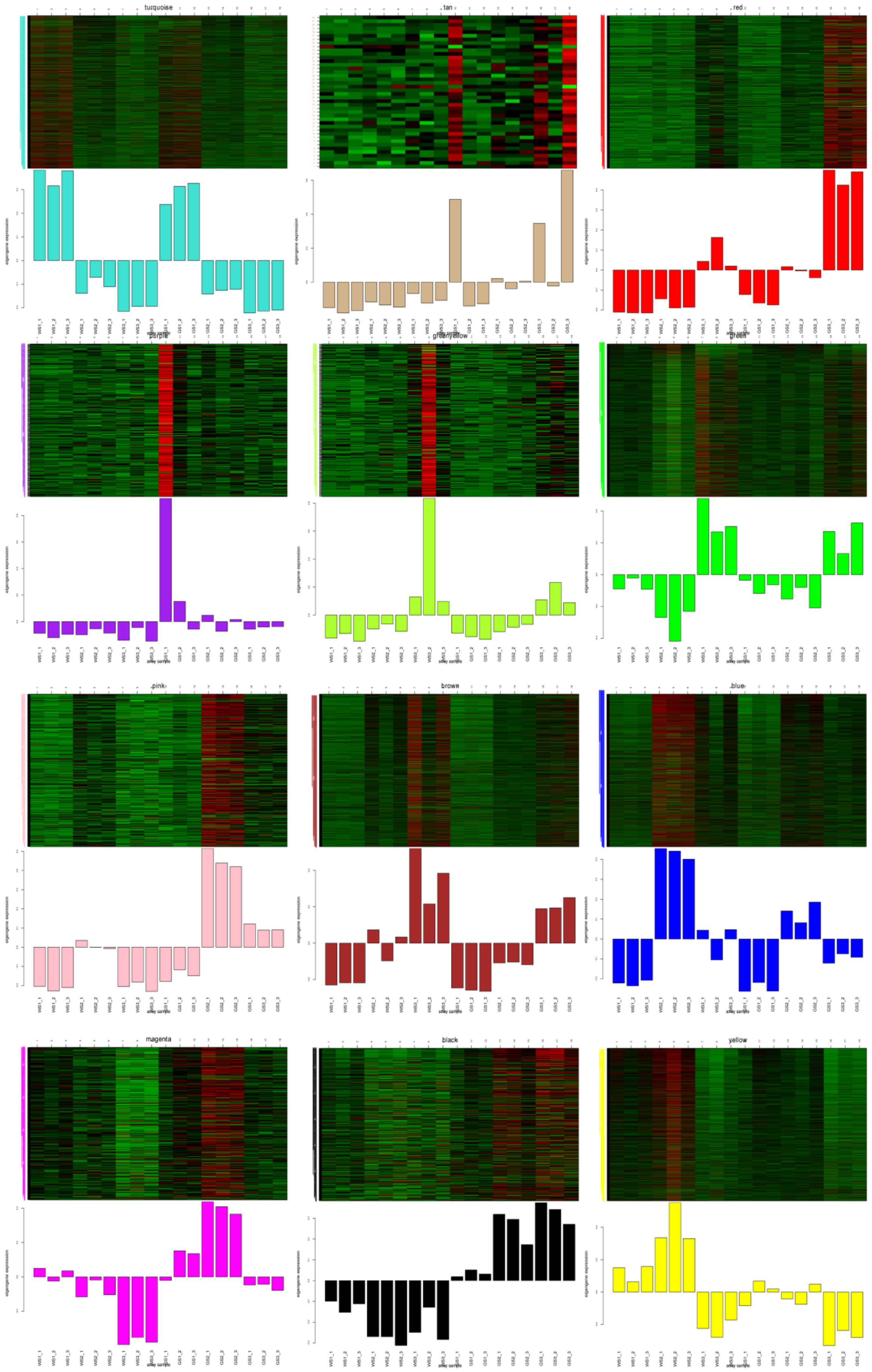

Figure S4 Expression pattern of eigengenes in 12 modules.

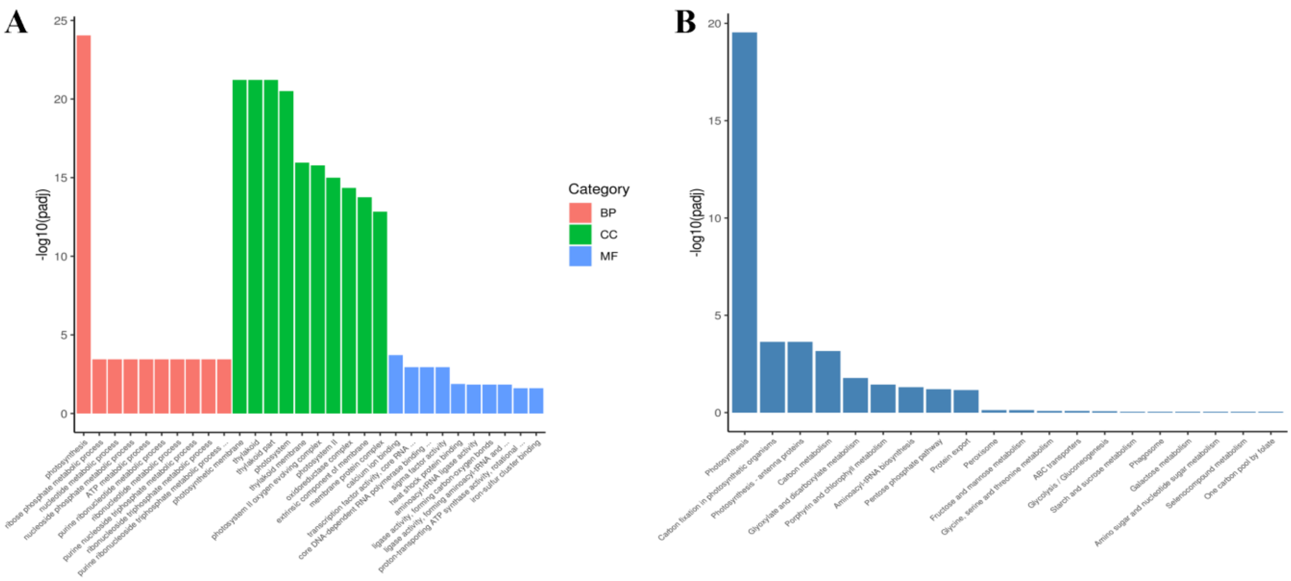

Figure S5 Functional classification of genes in black module. **(A)** GO functional classification of genes in black module. **(B)** KEGG functional classification of genes in black module

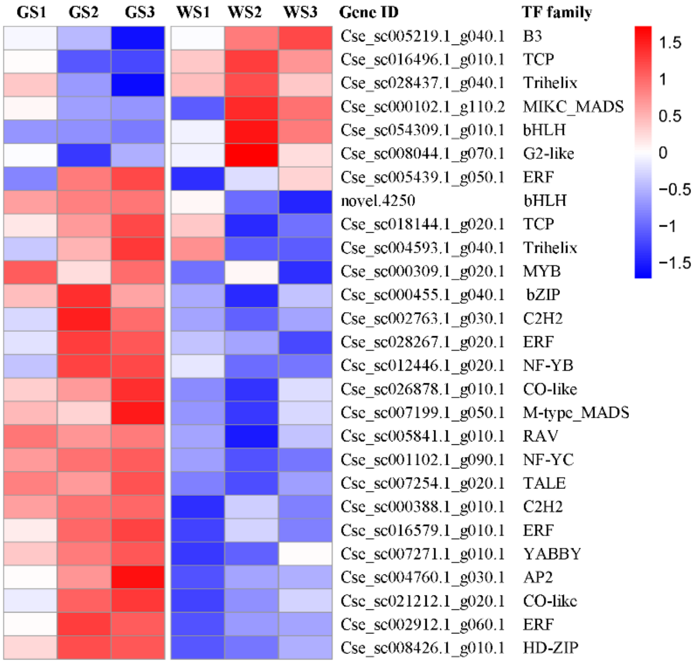

Figure S6 Heatmap of transcription factors in black module.
